# MicroRNA-203 suppresses cell proliferation and migration by targeting BIRC5 and LASP1 in human triple-negative breast cancer cells

**DOI:** 10.1186/1756-9966-31-58

**Published:** 2012-06-19

**Authors:** Chen Wang, Xiangqian Zheng, Chunyan Shen, Yurong Shi

**Affiliations:** 1 Department of Breast Oncology, Tianjin Medical University Cancer Hospital, Huanhuxi Ave, Tianjin, 300060, China; 2 Department of Thyroid and Neck Tumor, Tianjin Medical University Cancer Hospital, Huanhuxi Ave, Tianjin, 300060, China; 3 Department of Medical Oncology, Chinese Armed Police Medical Institute Affiliated Hospital, Tianjin, 300100, China; 4 Tianjin Cancer Institute, Huanhuxi Ave, Tianjin, 300060, China

**Keywords:** Triple-negative breast cancer, MiR-203, baculoviral IAP repeat-containing protein 5, Lim and SH3 domain protein 1, Proliferation, Migration

## Abstract

**Background:**

This study was performed to investigate the effect of microRNA-203 (miR-203) on cell proliferation and migration in triple-negative breast cancer (TNBC).

**Methods:**

Real-time PCR was performed to detect the expression of miR-203 in TNBC cell lines. miR-203 precursor and control microRNA (miRNA) were transfected into triple-negative breast cancer (TNBC) cell lines and the effects of miR-203 up-regulation on the proliferation and migration of cells were investigated. Meanwhile, the mRNA and protein levels of baculoviral IAP repeat-containing protein 5 (BIRC5) and Lim and SH3 domain protein 1 (LASP1) were measured. Luciferase assays were also performed to validate BIRC5 and LASP1 as miR-203 targets.

**Results:**

Both miR-203 and BIRC5 siRNA signicantly inhibited cell proliferation in TNBC cells. Both miR-203 and LASP1 siRNA signicantly inhibited cell migration in TNBC cells, also. Moreover, up-regulated of BIRC5 and LASP1 was able to abrogate the effects induced by transfection with the miR-203 precursor.

**Conclusions:**

These data suggest that miR-203 may function as a tumor suppressor in TNBC cells. Thus, miR-203 could be a potential therapeutic target for this disease.

## Background

Breast cancer is the most frequently diagnosed cancer and the leading cause of cancer death in women worldwide, accounting for 23% (1.38 million) of all new cancer cases and 14% (458,400) of all cancer deaths in 2008. Approximately half of all breast cancer cases and 60% of breast cancer-related deaths are estimated to occur in developing countries [1]. The large number of etiological factors and the complexity of breast cancer present challenge for prevention and treatment.

Triple-negative breast cancer (TNBC) is defined histologically as invasive carcinoma of the breast that lacks staining for estrogen receptor (ER), progesterone receptor (PgR), and the human epidermal growth factor receptor-2 (HER2). TNBC is associated with high proliferative rates, early recurrence, and poor survival rates. Much effort has been spent on the study of the biological behavior of TNBC cells to develop effective treatment strategies.

MicroRNAs (miRNAs) are small, non-coding RNAs of 19–25 nucleotides in length that are endogenously expressed in mammalian cells. miRNAs regulate gene expression post-transcriptionally, by pairing with complementary nucleotide sequences in the 3’-UTRs of specific target mRNAs [2,3]. This recently identified type of gene regulators is involved in modulating multiple cellular pathways, including cell proliferation, differentiation, and migration. Thus, miRNAs may function as oncogenic miRNAs or tumor suppressors [4-6]. Over 50% of miRNA genes are located in cancer-associated genomic regions [7]. The deletion or epigenetic silencing of a miRNA that normally represses the expression of one or more oncogenes might lead to carcinogenesis, tumor growth and invasion, as has been demonstrated for miR-200, miR-122 and miR-203 [8-10].

miR-203 is significantly down regulated in several cancers, including hepatocellular carcinoma [11], colon cancer [12], prostate cancer [13], and laryngeal cancer [14]. Because the down-regulated of miR-203 is common to a number of cancers, it has been hypothesized that miR-203 may play an important role in tumorigenesis and tumor development. However, the function of miR-203 in breast cancer remains unclear, especially in TNBC.

In this paper, we showed that miR-203 was down-regulated in TNBC cell lines and that the ectopic over-expression of miR-203 blocked tumor cell proliferation and migration in *vitro*. Furthermore, BIRC5 and LASP1 were identified as two direct functional targets of miR-203 in TNBC cells. These data suggest that the reduced expression of miR-203 facilitates the development and metastasis of TNBC.

## Materials and methods

### Cell culture and treatment

Human triple-negative breast cancer cell lines (MDA-MB-468 and MDA-MB-231) and normal breast cell line MCF-10A, were purchased from the American Type Culture Collection. MDA-MB-468 and MDA-MB-231 cells were maintained in DMEM (Gibco) supplemented with 10% FBS and 100 U/ml penicillin and 100 μg/ml streptomycin. MCF-10A cells were maintained in DMEM/F-12 supplemented with 10% FBS, insulin (10 μg /ml), hydrocortisone (500 ng/ml) and EGF (20 ng/ml). The cells were collected using 0.05% trypsin EDTA following the specified incubation period.

### Precursor miRNA/siRNA/plasmid transfection

Cells were seeded in 6-well plates at a concentration of 1 × 10^5^ and cultured in medium without antibiotics for approximately 24 h before transfection. Cells were transiently transfected with miR-203 precursor (Applied Biosystems) or negative control miRNA, BIRC5 siRNA (Sigma), LASP1 siRNA (Sigma) or control siRNA at a final concentration of 200nM. PcDNA-BIRC5 or pcDNA-LASP1 plasmid was also transfected into MDA-MB-231 cells using Lipofectamine 2000 (Invitrogen) according to the manufacturer’s protocol.

### Real-time PCR assay

Total RNA was extracted from cultured cells using the TRIzol reagent (Invitrogen). cDNA was obtained by reverse transcription of total RNA using a TaqMan Reverse Transcription Kit (Applied Biosystems) and iScript cDNA Synthesis kit (BIO-RAD), respectively. The expression level of mature miR-203 was measured using a TaqMan miRNA assay (Applied Biosystems) according to the provided protocol and using U6 small nuclear RNA as an internal control. Expression of BIRC5 and LASP1mRNA was detected using Power SYBR Green kit (Applied Biosystems). All experiments were performed in triplicate.

### Colony formation assay

Cells were seeded into a 12-well cell culture plate and incubated for 2 weeks at 37 °C after treatment. Then, cells were washed twice with PBS, fixed with cold methanol, stained with 0.1% crystal violet, washed and air dried.

### Migration assay

Cells were harvested and re-suspended in serum-free DMEM medium. For the migration assay, 5 × 10^4^ cells were added into the upper chamber of the insert (BD Bioscience, 8 μm pore size). Cells were plated in medium without serum, and medium containing 10% fetal bovine serum in the lower chamber served as the chemoattractant. After 6 h of incubation, cells were fixed with 3.7% formaldehyde and stained with crystal violet staining solution, and cells on the upper side of the insert were removed with a cotton swab. The migratory capacity was evaluated as the total number of cells on the lower surface of the membrane, as determined by microscopy.

### Western blot analysis

The cells in each well, including dead cells floating in the medium, were harvested and lysed in RIPA buffer. The protein concentrations of the lysates were determined using a bicinchoninic acid protein assay kit (Pierce Biotech). An aliquot of the lysate containing 50 μg proteins was subjected to sodium dodecyl sulfate polyacrylamide gel electrophoresis and then transferred to polyvinylidene fluoride membranes. The membranes were blocked with blocking buffer (TBST containing 5% non-fat milk) for 1 h at room temperature and then incubated overnight at 4 °C with the following specific primary antibodies: BIRC5, LASP1 β-actin (Cell Signaling Technology). Subsequent incubation with the appropriate horseradish peroxidase-conjugated secondary antibodies was performed for 2 h at room temperature. Signals were detected using enhanced chemiluminescence reagents (Thermo).

### Luciferase reporter assay

To evaluate the function of miR-203, the 3’-UTRs of BIRC5 and LASP1 with a miR-203 targeting sequence were cloned into the pMIR-REPORT luciferase reporter vector (Ambion). The sequences used to amplify BIRC5 3’-UTR were 5’-AAAGCCGGCCTGAAGTCTGGCGTAAGATG-3’ (forward) and 5’-GGACTAGTCCACATGAGACTTTATTG-3’ (reverse). The sequences used to amplify LASP1 3’-UTR were 5’-AAAGCCGGCGTCTTCTCTACAGTTCAC -3’ (forward) and 5’-GGACTAGTCCAGGAGAAAGATTCACTTG-3’ (reverse). Mutant BIRC5 and LASP1 3’-UTRs bearing a substitution of three nucleotides (TTT to CCC) in the miR-203 target sequence were generated using a Site-Directed Mutagenesis Kit (Agilent Technologies). Cells were co-transfected with luciferase reporter plasmids and miR-203 precursor (or control miRNA) along with Renilla Luciferase phRG-TK (Promega) as an internal control using Lipofectamine 2000 (Invitrogen). Luciferase activity was measured 72 h after transfection using the Dual-Luciferase Reporter Assay System (Promega). All experiments were performed in triplicate.

### Statistical analysis

Statistical analysis was performed using one-way ANOVA or Student’s t test. Values of *P* < 0.05 were considered significant. Data were represented as the mean ± S.D. GraphPad Prism 5.0 software was used for all data analysis.

## Results

### miR-203 expression was decreased in TNBC cell lines while BIRC5 and LASP1 expression was increased

**Figure 1:**
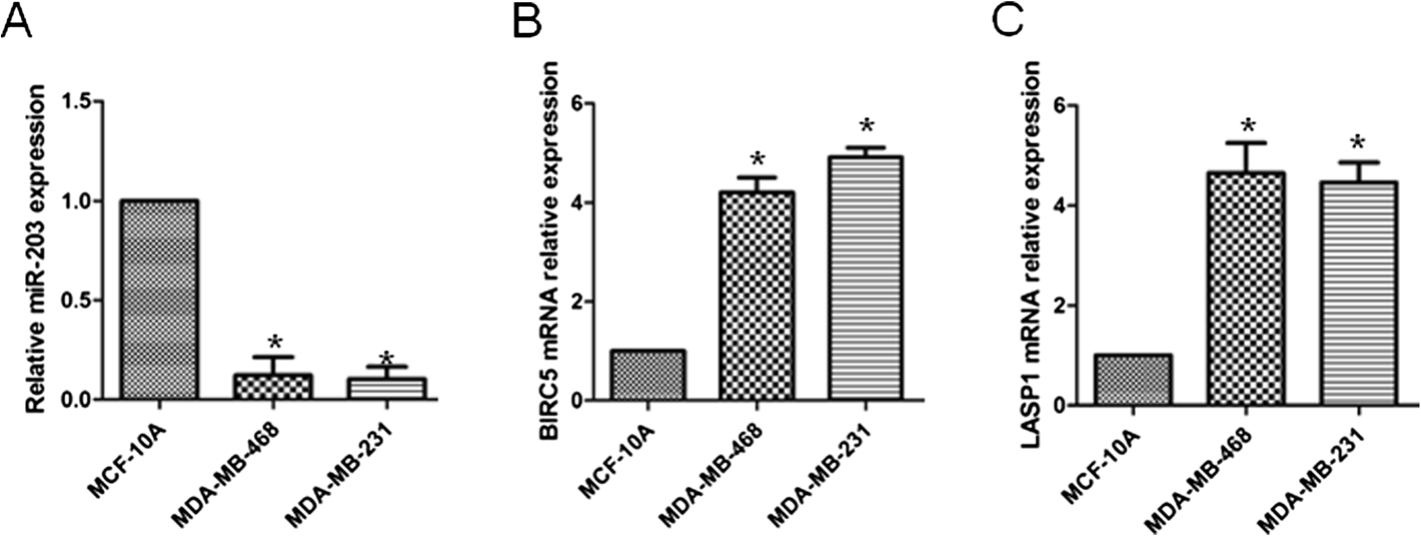
**miR-203 was down-regulated in TNBC cell lines while BIRC5 and LASP1 expression was up-regulated.** (A) Relative miR-203 expression was examined in the indicated breast cancer cell lines and the MCF-10A cell line. (B) Relative BIRC5 expression at mRNA level was examined in the indicated breast cancer cell lines and the MCF-10A cell line. (C) Relative LASP1 expression at mRNA level was examined in the indicated breast cancer cell lines and the MCF-10A cell line. miR-203 expression was normalized to that of U6 in each sample. BIRC5 and LASP1 mRNA expression was normalized to that of β-actin in each sample. *, *P* < 0.05.

We detected the abundance of miR-203 in triple-negative human breast cancer cell lines: MDA-MB-468 and MDA-MB-231 and a normal breast cell line: MCF-10A, by real-time PCR. TNBC cell lines (MDA-MB-468 and MDA-MB-231) showed significant miR-203 repression than normal breast cell line MCF-10A. We also detected BIRC5 and LASP1 expression at mRNA level in breast cancer cell lines and MCF-10A cell line. It was intriguing that in sharp contrast to the down-regulation of miR-203 in TNBC cells, BIRC5 and LASP1 expression is increased in TNBC cell lines compared to that in MCF-10A (Figure 1B&C). These data may implicate miR-203 expression is negatively correlated with BIRC5 and LASP1.

### miR-203 inhibited proliferation and migration of TNBC cells

**Figure 2:**
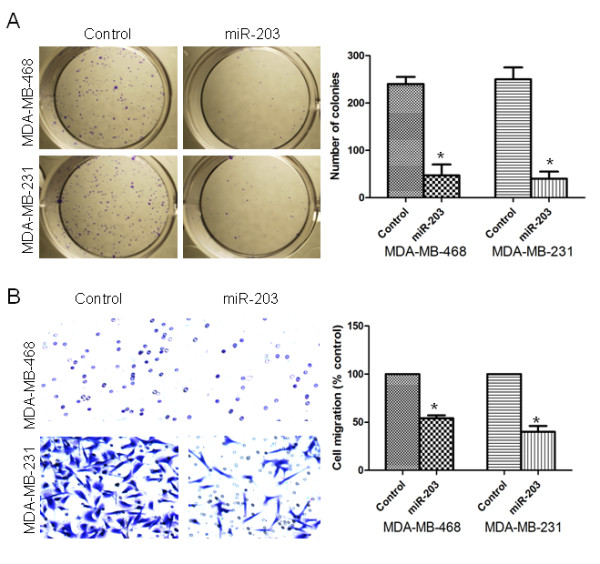
**miR-203 inhibited proliferation and migration of TNBC cells.** (A) The colony formation assay was used to measure cell proliferation capacity in MDA-MB-468 and MDA-MB-231 cells treated with control miRNA or miR-203 precursor. (B) A transwell migration assay was performed to detect the migratory capacity of MDA-MB-468 and MDA-MB-231 cells. *, *P* < 0.05.

Previous reports have shown that the over-expression of miR-203 has an impact on growth in prostate and laryngeal cancer cell lines [13,14]. Therefore, we investigated the effect of miR-203 on the proliferation of TNBC cells. Colony formation assay showed that a statistically significant inhibition of TNBC cell proliferation occurred after treatment with the miR-203 precursor (Figure 2A). To investigate whether miR-203 inhibits the migration of TNBC cells, we performed a transwell migration assay. Interestingly, the over-expression of miR-203 repressed the migration of the MDA-MB-231 and MDA-MB-468 cells. Cell mobility was significantly decreased by approximately 50% in miR-203-transfected cells compared with the control miRNA-transfected cells (Figure 2B). These observations suggest that miR-203 over-expression suppresses the mobility of TNBC cells in vitro.

### miR-203 post-transcriptionally down regulates BIRC5 and LASP1 expression by targeting the 3’-UTR regions of BIRC5 and LASP1

**Figure 3:**
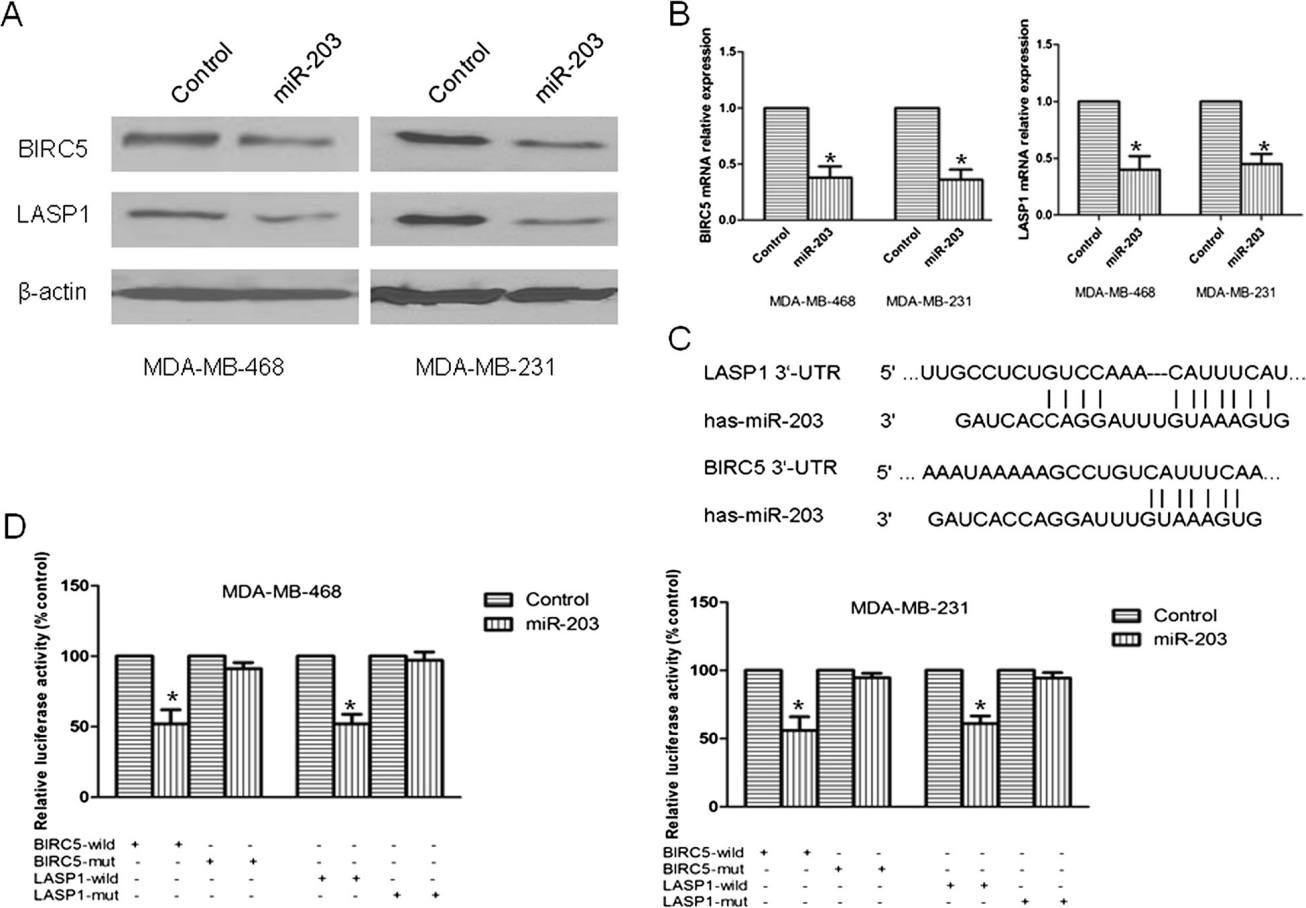
**BIRC5 and LASP1 were identified as miR-203 target genes.** (A) Immunoblots of BIRC5 and LASP1 protein in TNBC cells after treated with miR-203 precursor or control miRNA. β-actin was used as a loading control. (B) Relative BIRC5 and LASP1 expression at mRNA level in TNBC cells transfected with miR-203 precursor or control miRNA. The mRNA expression was normalized to that of β-actin. (C) Sequence alignment of miR-203 and its putative conserved target site in BIRC5 and LASP1 3’-UTR (downloaded from TargetScan 6.0). (D) Luciferase reporter assays of the interaction between miR-203 and the BIRC5 and LASP1 3’-UTRs. Assays were performed by co-transfection of miR-203 precursor with a luciferase reporter gene linked to the 3’-UTRs of BIRC5 and LASP1, containing either wild type or mutated miR-203 complementary sites. *, *P* < 0.05.

To explore the molecular mechanism of miR-203 activity, we used TargetScan 6.0 to search for target genes of miR-203, especially for genes with potential roles in promoting tumor cell proliferation and migration. It has been reported that individual miRNAs are capable of regulating dozens of distinct mRNAs. Based on this rationale, we selected two candidate miR-203 targets, BIRC5 and LASP1, for further study. We examined the influence of miR-203 on the endogenous expression of BIRC5 and LASP1 proteins by western blot. Intriguingly, BIRC5 and LASP1 expression were significantly decreased in miR-203-transfected MDA-MB-231 and MDA-MB-468 cells compared with control miRNA-transfected cells (Figure 3A). It was reported that miRNA can cause either mRNA degradation or translation repression. QPCR assay was also carried out to detect BIRC5 and LASP1 expression at mRNA level after transefected with miR-203 precursor in TNBC cells. We found that a decrease of BIRC5 and LASP1 mRNA in TNBC cells after treated (Figure 3B), so we believe that miRNA-203 regulates BIRC5 and LASP1 expression at both protein and mRNA levels. Moreover, a potential miR-203 targeting site was predicted in the 3’-UTRs of BIRC5 and LASP1 by TargetScan 6.0 (Figure 3C). To investigate whether the 3’-UTRs of BIRC5 and LASP1 are functional targets of miR-203 in breast cancer cells, we co-transfected the miR-203 precursor (or control miRNA) and pMIR-BIRC5-3’-UTR plasmid (or mutant) or pMIR-LASP1-3’-UTR plasmid (or mutant) into cells. Co-transfection with the miR-203 precursor was found to decrease wild type BIRC5 and LASP1 3’-UTR reporter activity (*P <* 0.05) compared with co-transfection with control miRNA in both two cell lines. However, co-transfection with the miR-203 precursor did not significantly alter mutant BIRC5 or LASP1 3’-UTR reporter activity (Figure 3D). These results demonstrated that miR-203 targets the predicted site within the 3’-UTRs of BIRC5 and LASP1 mRNA in TNBC cell lines.

### Repressing BIRC5 expression could inhibit the proliferation of MDA-MB-231 cells

**Figure 4:**
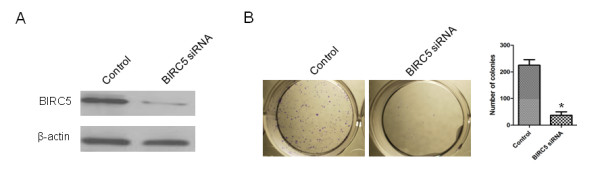
**Repressing BIRC5 expression could inhibit the proliferation of MDA-MB-231 cells.** (A) Immunoblots of BIRC5 protein in MDA-MB-231 cells treated with control siRNA or BIRC5 siRNA. β-actin was used as a loading control. (B) Colony formation assay was used to measure cell proliferative capacity in MDA-MB-231 cells treated with control siRNA or BIRC5 siRNA. *, *P* < 0.05.

To investigate the effect of BIRC5 on the proliferation of TNBC cell, we employed MDA-MB-231 cells as the model system to perform the subsequent studies. We evaluated the cell proliferative capacity of MDA-MB-231 cells transfected with BIRC5 siRNA (or control siRNA). The expression of BIRC5 protein in the cells transfected with BIRC5 siRNA was significantly decreased in comparison with that of cells transfected with control siRNA (Figure 4A), indicating that the expression of BIRC5 was effectively inhibited by BIRC5 siRNA. Subsequent studies showed that the proliferative capacity of cells transfected with BIRC5 siRNA was significantly lower than that of cells treated with control siRNA (Figure 4B).

### Repressing LASP1 expression could inhibit migration of MDA-MB-231 cells

**Figure 5:**
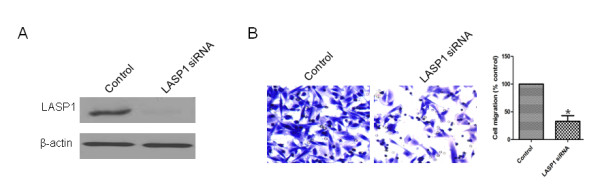
**Repressing LASP1 expression could inhibit migration of MDA-MB-231 cells.** (A) Immunoblots of LASP1 protein in MDA-MB-231 cells treated with control siRNA or LASP1 siRNA. β-actin was used as a loading control. (B) Transwell migration assay was performed to detect the migratory capacity of MDA-MB-231 cells treated with control siRNA or LASP1 siRNA. *, *P* < 0.05.

To investigate the effect of LASP1 on the migration of TNBC cell, we evaluated the cell migratory capacity of MDA-MB-231 cells transfected with LASP1 siRNA (or control siRNA). The expression of LASP1 protein in the cells transfected with LASP1 siRNA was significantly decreased in comparison with that of cells transfected with control siRNA (Figure 5A), indicating that the expression of LASP1 was effectively inhibited by LASP1 siRNA. Subsequent studies showed that the migratory capacity of cells transfected with LASP1 siRNA was significantly lower than that of cells treated with control siRNA (Figure 5B).

### The inhibition of MDA-MB-231 cell proliferation by miR-203 is attenuated by the over-expression of BIRC5

**Figure 6:**
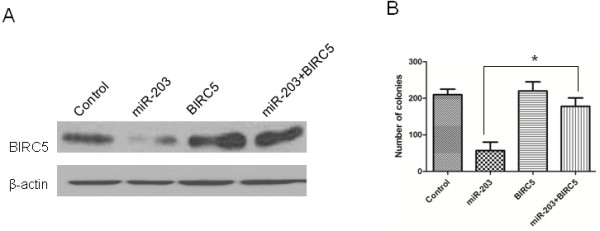
**Over-expression of BIRC5 could significantly attenuate the effect of miR-203 on the inhibition of MDA-MB-231 cell proliferation.** (A) BIRC5 protein expression was detected by western blot and normalized to β-actin protein levels. (B) Colony formation assay was performed to detect proliferative capacity in MDA-MB-231 cells. *, *P* < 0.05.

To provide direct evidence that down-regulation of BIRC5 is required for the anti-tumorigenic effects of miR-203, we transfected MDA-MB-231 cells with pcDNA-BIRC5 and miR-203 precursor. We first confirmed that BIRC5 and miR-203 have been conducted into the cells (Figure 6A), then, we used colony formation assay to show that the inhibition of MDA-MB-231 cell proliferation by miR-203 could be partially rescued by BIRC5 up-regulated (Figure 6B). These data clearly indicate that the ectopic over-expression of BIRC5 could efficiently block the effect on proliferation caused by miR-203.

### The inhibition of MDA-MB-231 cell migration by miR-203 is attenuated by the over-expression of LASP1

**Figure 7:**
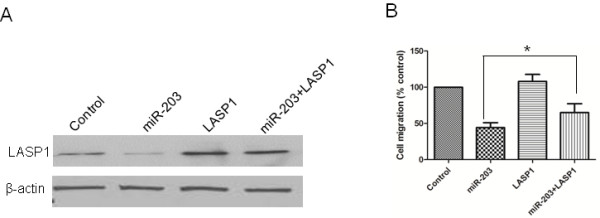
**Over-expression of LASP1 significantly attenuated the effect of miR-203 on the inhibition of MDA-MB-231 cell migration.** (A) LASP1 protein expression was detected by western blot and normalized to the levels of β-actin protein. (B) Transwell migration assay was performed to detect the migratory capacity of MDA-MB-231 cells. *, *P* < 0.05.

To provide direct evidence that miR-203 inhibits the migration of TNBC cells through the LASP1-mediated signal pathway, we transfected MDA-MB-231 cells with miR-203 precursor and pcDNA-LASP1. We confirmed the effect of the transfection by western blot (Figure 7A). The migration assay showed that the over-expression of LASP1 could partially rescue the migratory capacity of MDA-MB-231 cells treated with the miR-203 precursor (Figure 7B).

## Discussion

The recent discovery of a class of small non-coding RNAs, called microRNAs, has received significant attention in cancer research [15,16]. The aberrant expression of oncogenic miRNAs is associated with the development and progression of many cancers, including breast cancer. Conversely, the over-expression of tumor suppressor miRNAs may repress cancer cell proliferation and migration, but the mechanisms by which miRNAs affect oncogenesis remain to be elucidated. In the present study, we showed that miR-203 is down-regulated in TNBC cell lines compared with the normal breast cell line. Moreover, we showed that the over-expression of miR-203 could suppress the proliferation and migration of TNBC cells, accompanied by a decrease in the expression of BIRC5 and LASP1, suggesting that miR-203 has tumor-suppressive effects in TNBC.

Consistent with our results, miR-203 expression is down regulated in several cancer cells, including liver cancer [11], prostate cancer [13], and some types of leukemia [9]. It was reported that forced miR-203 expression in esophageal cancer cell lines repressed ΔNP63 levels, inhibited cell growth and promoted apoptosis [17]. Taken together, these results suggest that miR-203 may act as a tumor suppressor and is down-regulated in cancer development. It has also reported that individual miRNAs are capable of regulating dozens of distinct mRNAs, so we considered the possibility that miRNA-203 might act on several target genes rather than a single target. We identified two potential miR-203 target genes: BIRC5 and LASP1.

BIRC5 is expressed during embryonic and fetal development but is undetectable in terminally differentiated normal adult tissue. However, it is re-expressed in human cancer cells at a frequency of 34-100% [18,19]. BIRC5 is a member of the IAP family of proteins that contain a single BIR domain and an extended C-terminal helical coiled-coil domain [20,21]. Up-regulation of BIRC5 is a frequent event in breast cancer, suggesting that BIRC5 may play an important role in tumorigenesis; furthermore, its expression in breast cancer tissue is significantly associated with poor clinical outcome [22-25]. It was reported that BIRC5 knockdown might inhibit proliferation and induce apoptosis in cancer cells [26]. Here, we used MDA-MB-231 as a TNBC cell model to demonstrate that repressing BIRC5 expression by siRNA could significantly inhibit the proliferation of TNBC cell lines, implying that BIRC5 played a positive role in TNBC cell proliferation. Moreover, we showed that BIRC5 over-expression could partially abrogate the proliferate inhibition induced by miR-203. This key observation indicates that the negative control of BIRC5 levels is a critical aspect of the tumor-suppressive activity of miR-203 in TNBC. Therefore, the identification of BIRC5 as a miR-203 target gene may explain, at least in part, the molecular mechanism of tumor suppression by miR-203.

LASP1 was initially identified in a cDNA library prepared from breast cancer metastases. The LASP1 protein includes three domains: an N-terminal LIM domain, a nebulin repeat domain and a C-terminal SH3 domain [27]. LASP1 is expressed at low basal levels in all normal human tissues, but is over-expressed in metastatic human breast cancer [28], ovarian cancer [29] and medulloblastoma [30]. Increased LASP1 expression could lead to a more aggressive breast carcinoma phenotype, and knocking down LASP1 may reduce the migratory capacity of breast cancer cells, possibly by influencing the localization of zyxin [29]. In our study, we identified the LASP1 transcript as a target of miR-203 in TNBC cells and found that inhibition of TNBC cell migratory capacity was accompanied by a reduction in LASP1 expression. We also showed that repressing LASP1 expression by siRNA could significantly inhibit the migration of MDA-MB-231 cells, implying that LASP1 played a positive role in TNBC cell migration. Moreover, we demonstrated that decreased LASP1 expression is essential for the miR-203-mediated inhibition of TNBC cell migration, showing that the over-expression of LASP1 could partially rescue the migration inhibition induced by miR-203 in MDA-MB-231 cells.

In conclusion, our data suggest that miR-203 could inhibit the proliferation and migration of TNBC cells by directly regulating the expression of BIRC5 and LASP1. Moreover, the activation of miR-203 may be a potentially useful novel strategy for inhibiting TNBC growth and metastasis.

## Competing interests

The authors declare that they have no competing interests.

## Authors’ contributions

CW performed the miRNA, siRNA and plasmid transfection, the reporter gene construction, the luciferase experiments and drafted the manuscript. XQZ performed the western blot experiments. CYS performed the qRT-PCR. YRS performed colony formation assay and the migration assay. All authors read and approved the final manuscript.
